# Supplementary material for: Effectiveness of physical activity on immunity markers and quality of life in cancer patient: a systematic review
Source: PeerJ. 2022 Aug 2;10:e13664. doi: 10.7717/peerj.13664 (PMC9354736; doi:10.7717/peerj.13664)
Supplement: File S1 [file peerj-10-13664-s001.docx]

**Table S1.** Sources of information and search strategies.

| **Database** | **Search Strategies** | **Results** |
| --- | --- | --- |
| **PubMed** | (((Neoplasm OR Cancer OR Malignancy OR Malignant Neoplasm OR tumor) AND (Immunology OR Immunity OR Lymphocytes OR Natural killer cells)) AND (Physical Activity OR Exercise OR Aerobic Exercise OR Exercise training)) AND (Quality of life OR Life Quality OR Related Quality Of Life OR HRQOL) | 308 |
| **Scopus** | neoplasm OR cancer OR malignancy OR malignant AND neoplasm OR tumor AND immunology OR immunity OR lymphocytes OR natural AND killer AND cells AND physical AND activity OR exercise OR aerobic AND exercise OR exercise AND training AND quality AND of AND life OR life AND quality OR related AND quality AND of AND life OR hrqol | 641 |
| **Cochrane** | Neoplasm OR Cancer OR Malignancy OR Malignant Neoplasm OR tumor AND Immunology OR Immunity OR Lymphocytes OR Natural killer cells AND Physical Activity OR Exercise OR Aerobic Exercise OR Exercise training AND Quality of life OR Life Quality OR Related Quality Of Life OR HRQOL | 266 |
| **ScienceDirect** | (Physical Activity OR Exercise) AND (Quality of life OR QOL) AND (Cancer OR Cancers) AND (Immunity) | 12716 |

**Table S2.** Quality of included studies by JBI critical appraisal checklist for studies reporting prevalence data.

| **Study name** | | **Question** | | | | | | | | | **Overall** |
| --- | --- | --- | --- | --- | --- | --- | --- | --- | --- | --- | --- |
|  |  | 1 | 2 | 3 | 4 | 5 | 6 | 7 | 8 | 9 |  |
| 1 | Chasen et al., 2013 | Yes | Yes | Yes | Yes | Yes | Yes | Yes | Yes | Yes | 9 |
| 2 | Ergun et al., 2013 | Yes | Yes | Yes | Yes | Yes | Yes | Yes | Yes | Yes | 9 |
| 3 | Sprod et al., 2010 | Yes | Yes | Yes | Yes | Yes | Yes | Yes | Yes | Yes | 9 |
| 4 | Kim et al., 2017 | Yes | Yes | Yes | Yes | Yes | Yes | Yes | Yes | Yes | 9 |
| 5 | Hojan et al., 2016 | Yes | Yes | Yes | Yes | Yes | Yes | Yes | Yes | Yes | 9 |
| 6 | Hojan et al., 2017 | Yes | Yes | Yes | Yes | Yes | Yes | Yes | Yes | Yes | 9 |
| 7 | Roberge et al., 2020 | Yes | Yes | Yes | Yes | Yes | Yes | Yes | Yes | Yes | 9 |
| 8 | Baumann et al., 2010 | Yes | Yes | Yes | Yes | Yes | Yes | Yes | Yes | Yes | 9 |
| 9 | Galvao et al., 2010 | Yes | Yes | Yes | Yes | Yes | Yes | Yes | Yes | Yes | 9 |

(1) appropriate sampling frame, (2) proper sampling technique, (3) adequate sample size, (4) study subject and setting description, (5) sufficient data analysis, (6) use of valid methods for the identified conditions, (7) valid measurement for all the participants, (8) using appropriate statistical analysis, and (9) adequate response rate.

**Table S3:** Summary of finding using GRADE quality assessment.

| **Outcome** | **Certainty assessment** | | | | | | | **№ of patients** | | **Effect** | **Certainty** | |
| --- | --- | --- | --- | --- | --- | --- | --- | --- | --- | --- | --- | --- |
|  | **№ of studies** | **Study design** | **Risk of bias** | **Inconsistency** | **Indirectness** | **Imprecision** | **Other considerations** | **Intervention group** | **Control group** | **Absolute (95% CI)** |  |  |
| C-reactive protein | 3 | RCT | Not serious | Very serious^b^ | Not serious | serious^a^ | None | 106 | 38 | SMD 0.78 lower (0.92 higher to 0.56 lower) | ⨁◯◯◯ Very low |  |
| Interleukin-6 | 3 | RCT | Not serious | Very serious^b^ | Not serious | serious^a^ | None | 56 | 56 | SMD 0.02 higher (0.57 lower to 0.62 higher) | ⨁◯◯◯ Very low |  |
| Interleukin-1B | 3 | RCT | Not serious | Very serious^b^ | Not serious | serious^a^ | None | 72 | 68 | SMD 0.40 lower (0.31 lower to 0.6 higher) | ⨁◯◯◯ Very low |  |
| Tumour necrosis factor alpha | 4 | 3 RCT  1 Pilot study | Not serious | Very serious^b^ | Not serious | serious^a^ | None | 141 | 77 | SMD 0.76 SD lower (0.66 lower to 0.97 higher) | ⨁◯◯◯ Very low |  |
| Interleukin-8 | 1 | Pilot study | Not serious | Very serious^b^ | Not serious | serious^a^ | None | 60 | - | SMD 0.81 SD lower (0.51 lower to 0.84 higher) | ⨁◯◯◯ Very low |  |
| Natural killer cell | 1 | Exp. study | Not serious | Very serious^b^ | Not serious | serious^a^ | None | 22 | 21 | SMD 0.34 SD lower (0.26 lower to 0.57 higher) | ⨁◯◯◯ Very low |  |
| Fatigue | 5 | 4 RCT  1 Exp. study | Not serious | Very serious^b^ | Not serious | serious^a^ | None | 161 | 89 | SMD 0.72 lower (0.34 lower to 0.57 lower) | ⨁◯◯◯ Very low |  |
| Quality of Life | 9 | 6 RCT  1 Exp. study 1 Pilot study | Not serious | Very serious^b^ | Not serious | serious^a^ | None | 301 | 168 | SMD 0.55 lower (0.87 lower to 0.23 lower) | ⨁◯◯◯ Very low |  |

a: the included studies recorded a small sample size for both the control and intervention groups.

b: there is considerable heterogeneity in the studies,
