# Supplementary material for: Effectiveness of physical activity on immunity markers and quality of life in cancer patient: a systematic review
Source: PeerJ. 2022 Aug 2;10:e13664. doi: 10.7717/peerj.13664 (PMC9354736; doi:10.7717/peerj.13664)
Supplement: File S2 [file peerj-10-13664-s002.docx]

- 1. ***Justification***

Exercise therapy is becoming more important in the rehabilitation of cancer patients because it is thought to modulate immunity and inflammation. There is a need for more knowledge and information on the effects of exercise on immune function in these patients (Hojan et al., 2016). Moreover, Exercise appears to have the most positive effects on host defence and disease vulnerability or severity in an individual with a suppressed immune function. Exercise may play an important role in the defence against tumour cells by improving Natural Killer cytotoxic activity and the function of neutrophils (Koelwyn et al., 2020). In addition, although it is established that physical activity has anti-inflammatory benefits in healthy individuals, its anti-inflammatory effects in cancer patients suffering from cancer-related tiredness have not yet been identified (Hojan et al., 2016).

On the other hand, physical activity may improve the quality of life in cancer patients by reducing fatigue-related daily interference, however, the relationship between changes in physical activity and these psychosocial aspects during therapy is not well understood (Thornton et al., 2010). therefore Physical activity may help to reduce the severity of the physical and psychological effects of cancer diagnosis and treatment (Mardani et al., 2021). Hence this systematic review examines changes in immunological markers and QOL in cancer patients following physical activity intervention.

- 1. ***What is the rationale of the study?***

Physical activity is associated with short-term immune-boosting and long-term anti-inflammatory effects. we found that Natural Killer cytotoxic activity increased after exercise in cancer patients, along with lymphocyte proliferation. We found that a significant reduction in C-reactive protein in response to exercise. Therefore, regular exercise has improved QOL, reduced depression and improved immunity markers in cancer patients. Before the diagnosis, physically active patients should continue their exercise regimens, while those who live a sedentary lifestyle should commence a safe, moderate exercise program. More studies are needed to better understand the mechanism between exercise and immune function in cancer patients as well as to emphasize improved QOL on clinical outcomes among cancer patients.

**References:**

HOJAN, K., KWIATKOWSKA-BOROWCZYK, E., LEPOROWSKA, E., GÓRECKI, M., OZGA-MAJCHRZAK, O., MILECKI, T. & MILECKI, P. J. E. J. P. R. M. 2016. Physical exercise for functional capacity, blood immune function, fatigue, and quality of life in high-risk prostate cancer patients during radiotherapy: a prospective, randomized clinical study. 52**,** 489-501.

KOELWYN, G. J., ZHUANG, X., TAMMELA, T., SCHIETINGER, A. & JONES, L. W. J. N. M. 2020. Exercise and immunometabolic regulation in cancer. 2**,** 849-857.

MARDANI, A., PEDRAM RAZI, S., MAZAHERI, R., HAGHANI, S. & VAISMORADI, M. J. I. J. O. N. P. 2021. Effect of the exercise programme on the quality of life of prostate cancer survivors: A randomized controlled trial. 27**,** e12883.

THORNTON, L. M., ANDERSEN, B. L. & BLAKELY, W. P. J. H. P. 2010. The pain, depression, and fatigue symptom cluster in advanced breast cancer: Covariation with the hypothalamic–pituitary–adrenal axis and the sympathetic nervous system. 29**,** 333.
